# Cost-effectiveness and cost utility analysis of three pneumococcal conjugate vaccines in children of Peru

**DOI:** 10.1186/1471-2458-13-1025

**Published:** 2013-10-30

**Authors:** Jorge Alberto Gomez, Juan Carlos Tirado, Aldo Amador Navarro Rojas, Maria Mercedes Castrejon Alba, Oleksandr Topachevskyi

**Affiliations:** 1 GSK Argentina, Victoria, Buenos Aires, Argentina; 2 Complejo Hospitalario San Pablo, Lima, Peru; 3 GSK Peru, Lima, Peru; 4 GSK Panama, Panama City, Panama; 5 GSK Belgium, Wavre, Belgium; 6 Carlos Casares, 3690 – B1644CD, Victoria, Buenos Aires, Argentina

**Keywords:** Pneumococcal disease, Pneumococcal vaccines, Cost-effectiveness, Acute otitis media, Non-typeable *Haemophilus influenzae*, Peru

## Abstract

**Background:**

The clinical and economic burden associated with invasive and non-invasive pneumococcal and non-typeable *Haemophilus influenzae* (NTHi) diseases is substantial in the Latin America and Caribbean region, where pneumococcal vaccines have only been introduced to a few countries. This study analyzed the cost-effectiveness and cost utility of three different pneumococcal conjugate vaccines (PCVs) for Peru.

**Methods:**

A Markov model that simulated the disease processes in a birth cohort over a lifetime, within 1,128 month cycles was used to evaluate the cost-effectiveness of 10-valent pneumococcal NTHi protein D conjugate vaccine (PHiD-CV) and 7- and 13-valent PCVs (PCV-7 and PCV-13). Expected quality-adjusted life years (QALYs), cost-savings and incremental cost-effectiveness ratios (ICERs) were calculated.

**Results:**

Without vaccination, pneumonia was associated with the greatest health economic burden (90% of QALYs lost and 63% of lifetime direct medical costs); while acute otitis media (AOM) was responsible for 1% of QALYs lost and 25% of direct medical costs. All vaccines were predicted to be cost-effective for Peru, with PHiD-CV being most cost-effective. PHiD-CV was predicted to generate 50 more QALYs gained and required a reduced investment (−US$ 3.4 million) versus PCV-13 (discounted data), and was therefore dominant and cost saving. The probabilistic sensitivity analysis showed that PHiD-CV generated more QALYs gained at a reduced cost than PCV-13 in 84% of the simulations and less QALYs gains at a reduced cost in 16%. Additional scenarios using different assumptions on vaccine efficacies based on previous evidence were explored, but no significant change in the overall cost-effective results were observed.

**Conclusions:**

The results of this modeling study predict that PCVs are likely to be a cost-effective strategy to help relieve the epidemiological and economic burden associated with pediatric pneumococcal and NTHi diseases for Peru. PHiD-CV is likely to be a dominant (better health gains at a reduced net cost) intervention compared to PCV-13 or PCV-7. The most significant drivers for these results are the better health and economic profile of PHiD-CV against AOM and its reduced cost per dose available through the PAHO Revolving Fund in the LAC region.

## Background

*Streptococcus pneumoniae* is one of the leading causes of morbidity and mortality in Latin American children. O’Brien et al. reported that, in the year 2000, *S. pneumoniae* infections led to 648,000 cases and 24,300 deaths due to pneumonia, 9,500 cases and 4,500 deaths due to meningitis, and 11,700 severe cases and 4,300 deaths of non-pneumonia, non-meningitis invasive pneumococcal disease (IPD) among children aged <5 years in the Americas World Health Organization (WHO) Region [1]. Furthermore, Valenzuela et al. estimated the annual burden of pneumococcal diseases to include 330,000 pneumonia cases, 1,200 cases of sepsis, 3,900 cases of meningitis, and 1.3 million cases of acute otitis media (AOM) among children aged <5 years in the Latin America and Caribbean (LAC) region [2]. The annual number of deaths due to pneumococcal disease was 12,000-28,000 [2].

Although a 7-valent pneumococcal conjugate vaccine (PCV-7) (Prevenar®*,* Pfizer, USA) has been licensed in the LAC region for several years, it has only recently been introduced into a few national immunization programs. Peru was one of the few LAC countries that introduced PCV-7, in 2009 [3], and is traditionally using the Pan American Health Organization (PAHO) revolving fund for its vaccination program [4]. However, it has been reported that the introduction of PCV-7 into national programs does not sufficiently reduce pneumococcal disease burden, because of the low vaccine coverage reached in these programs and the low number of pneumococcal serotypes covered by the vaccine [5,6].

New PCVs are now available against these diseases with broader serotype coverage. Firstly, PHiD-CV (Synflorix™, GlaxoSmithKline Biologicals, Belgium), a 10-valent pneumococcal non-typeable *Haemophilus influenzae* protein D conjugate vaccine that includes three additional serotypes (1, 5, and 7 F) compared to PCV-7 (serotype 4, 6B, 9 V, 14, 18C, 19 F and 23 F) and uses protein D from non-typeable *Haemophilus influenzae* (NTHi) as a carrier protein, which may offer additional protection against NTHi [7]. Secondly, PCV-13 (Prevenar 13®, Pfizer/Wyeth, USA), a PCV composed of six additional capsular *S. pneumoniae* polysaccharide serotypes (1, 3, 5, 6A, 7 F and 19A) compared to PCV-7, which are individually conjugated to non-toxic diphtheria as a carrier protein [8].

Reducing and eliminating vaccine-preventable diseases requires evidence-based and informed policy decision making. The National Institute of Health of Peru has recently completed a limited economic evaluation of the new pneumococcal vaccines, and concluded that PHiD-CV and PCV-13 would be more cost-effective than PCV-7 in terms of preventing pneumonia hospitalizations [9]. The aim of the current study is to provide comprehensive data on the comparative cost utility and cost-effectiveness analysis of the different PCVs in order to facilitate the decision-making process of immunization policy at a national level.

## Methods

### Modeling approach

An age-compartmental, deterministic and static cohort Markov model developed in MS Excel® (Microsoft Corporation, Redmond, WA) was used to assess the health and economic impact of PCVs in Peru. The model simulated the disease process of invasive diseases (ID), community acquired pneumonia (CAP), and AOM caused by *S. pneumoniae* and NTHi in a birth cohort over a lifetime with 1,128 month cycles (or 94 years) to calculate expected costs and quality-adjusted life years (QALYs) over time.

The impact of vaccination was assessed in a cohort (vaccinated or unvaccinated) that was followed over a lifetime, as previously described [10,11]. A direct vaccine effect was estimated as a reduction in the incidence of ID, CAP, and AOM. Six diseases (pneumococcal meningitis, NTHi meningitis, pneumococcal bacteremia, NTHi bacteremia, CAP, and AOM) were followed through specific disease outcomes and treatment pathways. The model compared different accumulated conditions over the lifetime of a birth cohort - unvaccinated and vaccinated with PCV-7, PCV-13, or PHiD-CV. Table 1 presents details of the base-case analysis [5,12,13].

**Table 1:** Assumptions and main parameters used in the base-case analysis

**Parameter**	**Assumptions and values used in the base case**
Country	Peru
Population	Newborn cohort of 2007 (500,700) [12]
Perspective	Payers of the healthcare system
Time horizon	Lifetime
Comparators	No vaccination, PCV-7, PCV-13, and PHiD-CV
Indirect effects	None considered in the base-case analysis
NTHi ID efficacy	35.3% for PHiD-CV [13]
Outcomes	ID: meningitis and bacteremia (including long-term sequelae for meningitis) Ambulatory and hospitalized Pneumonia AOM (including long-term sequelae and myringotomy)
Cross protection	Considered for 6A and 19A in ID
Vaccine coverage	95% of the cohort receives full schedule for PHiD-CV and PCV-13; PCV-7 was studied with 83% coverage [5]
Vaccination schedule	2 + 1, same for the 3 vaccines: two doses at 2 and 4 months of age and a booster dose at 12 months of age
Vaccine price	Vaccine prices per dose for PCV-13 (US$ 16.34) and PHiD-CV (US$ 14.24) were obtained from the PAHO Revolving Fund 2012, and PCV-7 (US$ 20.00) from the PAHO Revolving Fund 2010 (last year of availability)
Duration of immunity	9 years, with vaccine efficacy modeled as previously shown for all the outcomes evaluated [11]
Discounting	3.5% for costs and health events

AOM, acute otitis media; ID, invasive disease; NTHi, non-typeable *Haemophilus influenzae*; PAHO, Pan American Health Organization; PCV-7, 7-valent pneumococcal conjugate vaccine; PCV-13, 13-valent pneumococcal conjugate vaccine; PHiD-CV, 10-valent pneumococcal non-typeable Haemophilus influenzae protein D conjugate vaccine; WHO, World Health Organization.

### Model inputs

#### Epidemiological burden

The epidemiological data for ID and pneumonia (Additional file 1: Table S1, Additional file 1: Table S2 and Additional file 1: Table S3) used to populate the Markov model were taken from a previous study in six Latin American countries (Mexico, Brazil, Argentina, Chile, Colombia, and Peru) [14], which reviewed databases from the Ministry of Health and published and unpublished studies from Peru using the Delphi method [15].

Country-specific data on AOM were not available. Therefore, the average incidence rate from three Latin American AOM studies (carried out in Brazil, Mexico and Chile) [16-18] was used (Additional file 1: Table S4). This average showed a peak incidence rate of AOM during the first year of life (8,943.5 cases per 100,000 infants). In our simulation, 60% of the studied cohort will suffer from AOM that will impact to the health system. The incidence of tympanocentesis and sequelae were calculated by multiplying AOM incidence by the corresponding percentages provided by Delphi panels. The frequency of NTHi and *S. pneumoniae* in AOM was obtained from an international review (32.3% for NTHi and 35.9% for *S. pneumoniae*[19]) because country-specific epidemiological data for AOM were not available.

The frequency of NTHi-associated ID was calculated from data from the Latin American surveillance system (SIREVA) promoted by PAHO [20]: the ratio of *S. pneumoniae* to NTHi meningitis was 11:1 and *S. pneumoniae* to NTHi bacteremia was 17:1 (please see Additional file 1 for calculation).

*S. pneumoniae* serotype distribution was obtained from the Latin American Network for Surveillance of Pneumonia & Bacterial Meningitis Agents (SIREVA II). We used average data specifically reported for Peru for 2000–2006 (n = 222) (Additional file 1: Table S5) [21,22]. The epidemiological data used to calibrate the model for Peru was collected prior to the introduction of PCV-7 in 2009, as were all the other epidemiological data used. Peru reports to this network typing results of 17–50 cases per year. Although minor differences are reported in type distribution year by year, no clear trend was observed over time (even for serotypes 6A & 19A) and the annual sample size is not adequate to assure that the scenario of any individual year will represent a significant epidemiological change. Therefore, we decided to use average data of pneumococcal types distribution covering many years before vaccine introduction in the analysis.

#### Economic burden

Average costs of treatment for the different outcomes were estimated for Peru using micro-costing methods with unit costs from the three health sectors of Peru (public, private, and EsSalud), considering their reported coverage. Rates of service utilizations were obtained from Delphi panels [15,23], as previously described [14]. Costs (Additional file 1: Table S6) were obtained in 2009 Nuevos Soles and translated to 2009 US$ using the exchange rate for December 2009 (US$ 1 = 2.78 Nuevos Soles).

#### Vaccine efficacy assumptions

Vaccine efficacy varies with age as previously described [11]. Monthly cycles were chosen in order to estimate the effect of the vaccination precisely in the first 2 years of life. The vaccine is given at ages 2, 4, and 12 months. In order to account for partial early protection at ages 2 and 4 months, we subdivided the model into monthly cycles. It is important to estimate the effect of the vaccines in the <1-year age group precisely because the burden of disease amongst pediatric population is the highest in this age group. Vaccine efficacy input data for each of the vaccines assessed in this study were obtained or estimated from previously published papers [13,24-32] and abstracts [33-35] of clinical trials and a review [31]. The base-case analysis considers vaccine efficacy against *S. pneumoniae* related IPD and AOM, and against all-cause hospitalized pneumonia. We also assumed, in the base-case analysis, that the use of protein D from NTHi as a carrier protein in PHiD-CV may offer additional protection against NTHi disease (AOM & ID) [7,13]. Key model assumptions were validated with international pneumococcal vaccine and modeling experts during 2007–2008 (see Additional file 1 for details on experts consulted), and have been previously published [10,11].

#### Vaccine efficacy against ID

Vaccine efficacy against IPD was based on the serotype-specific efficacies reported for PCV-7 in a matched case–control study [24] and the serotype distribution of *S. pneumoniae* from SIREVA II [21,22] (Additional file 1: Table S5). Vaccine efficacy for the additional serotypes in the new vaccines was assumed to be the average of the vaccine efficacies observed for the serotypes included in PCV-7 (94.7%) [24]. The vaccine efficacy of PHiD-CV against NTHi-associated ID was assumed to be the same as that reported for NTHi-associated AOM (35.3%) [13]. This assumption was considered to be conservative as the vaccine efficacy of PCVs against ID is much higher than for mucosal diseases. For example, vaccine efficacy of PCV-7 against IPD (vaccine types) is ~95% [24], while its efficacy against mucosal diseases has been reported to be 58% (in pneumococcal AOM) [13,32].

Finally, similar cross-protection against *S. pneumoniae* serotypes 6A and 19A for ID was considered for PCV-7 and PHiD-CV. Immunogenicity studies have shown that PHiD-CV provides cross-reacting functional (opsonophagocytic) antibodies against 6A and 19A [36,37]. These studies have shown that PHiD-CV and PCV-7 provide similar levels of cross-functional antibodies against serotype 6A, but PHiD-CV provides higher levels of cross-reacting functional antibodies against serotype 19A than PCV-7. Cross-protection of PCVs against *S. pneumoniae* type 6A has been reported in several studies [24,32,38], and Hausdorff et al. have suggested that cross-protection against *S. pneumoniae* serotype 19A is likely [39]. In accordance with guidelines on the evaluation of new vaccines developed by the European Medicines Agency (EMEA) [40], in the absence of specific efficacy studies, immunogenicity data were considered and cross-protection levels of 76% against *S. pneumoniae* type 6A and 26% against *S. pneumoniae* type 19A for PCV-7 and PHiD-CV in ID were assumed, based on Whitney et al. [24].

#### Vaccine efficacy against CAP

Vaccine efficacy against CAP was based on the results of the recently completed COMPAS study, a Latin American trial of PHiD-CV [33-35]. Based on this study, vaccine efficacy against hospitalized all-cause pneumonia was assumed to be 23.4%, and against all-cause ambulatory pneumonia, 7.3% (similar for the three vaccines). These efficacies were used for PCV-7, PCV-13 and PHiD-CV, based on two PCV-7 studies [25,26], three studies of an experimental 9-valent PCV [27-29], a study with an experimental 11-valent conjugated vaccine [30], the COMPAS study of PHiD-CV [33-35], and a Cochrane Collaboration review [31], which show no clear relationship between vaccine efficacy against pneumonia and the serotypes contained in the PCV.

#### Vaccine efficacy against AOM

Vaccine efficacies of PCV-13, PCV-7, and PHiD-CV against AOM were extrapolated from a PCV-7 trial [32] and one of an 11-valent precursor to PHiD-CV [13]. Vaccine efficacy against AOM-related pneumococcal serotypes included in all three vaccines was assumed to be 58%, as previously shown [13,32]. Vaccine efficacy against AOM associated with the pneumococcal types not included in these vaccines was −33% (serotype replacement effect), as shown in the PCV-7 study [32]. Although PHiD-CV did not show any serotype replacement effect [13], it was included in the model for PHiD-CV following expert opinion. Vaccine efficacies against NTHi AOM were assumed to be 35.3% for PHiD-CV (based on the 11-valent precursor to PHiD-CV study [13]) and −11% for PCV-7 and PCV-13 (based on the PCV-7 study [32], incorporating a replacement effect as was found in this study). The vaccine coverage on the pneumococcal types isolated in AOM was calculated using the pneumococcal type prevalence in AOM described in a meta-analysis of AOM etiology in Latin America by Bardach et al. [41]. Overall, the final estimated coverages for PCV-7, PHiD-CV, and PCV-13 were 69.8%, 76.2%, and 89.5%, respectively.

#### Vaccine-related costs

Vaccine prices per dose were obtained from the 2012 PAHO Revolving Fund (RF) for PCV-13 (US$ 16.34) and PHiD-CV (US$ 14.24) (the first year with these two vaccines available at the PAHO RF), and from the PAHO Revolving Fund 2010 (the last year with this vaccine available at the PAHO RF) for PCV-7 (US$ 20.00). We decided to use the PAHO RF framework for the analysis because Peru and many other Latin American countries buy vaccines under this environment. Vaccine costs also included a 10% vaccine wastage cost and US$ 1 administrative costs per dose for either vaccine [42]. The base-case of the analysis was performed from a healthcare payer (Ministry of Health) perspective using direct medical costs.

### Cost-effectiveness analysis

Cost-effectiveness of the three vaccines (PCV-7, PCV-13, and PHiD-CV) was assessed against no vaccination, using a three-dose (2 + 1) vaccination schedule for each vaccine at 2, 4, and 12 months of age (Table 1). Vaccination coverage (three doses) of 83% was used for PCV-7, as reported by WHO Vaccine-Preventable Diseases Monitoring System for 2010 [5], and 95% was estimated for PHiD-CV and PCV-13 vaccines (Table 1).

The utility decrements (Additional file 1: Table S7) used for each outcome analyzed were obtained from international sources [43-46] due to lack of local data. A 3.5% discount rate for cost and health outcomes was applied to estimate the incremental cost-effectiveness ratios (ICERs). The gross domestic product (GDP) per capita (2009 US$) for the cost-effectiveness threshold was obtained from the International Monetary Fund (US$ 4,356 per capita for Peru) [47]. Cost-effectiveness thresholds of 1 or 3 GDP per capita were used, following the recommendations of the Commission on Macroeconomics and Health of the WHO [48,49].

### Sensitivity analyses

Univariate sensitivity analysis was completed for all of the model input parameters. Each parameter was varied up and down from the base-case value, and for each change, the ICER was re-estimated and compared to the cost-effectiveness threshold. Results were considered robust when ICER variations did not modify interpretation of the cost-effectiveness of vaccination. The list of model parameters and their associated sampling uncertainty included in the 1-way sensitivity analysis are shown in Additional file 1: Table S8.

Probabilistic sensitivity analysis (PSA) was performed by recording the results of 1,000 Monte Carlo simulations, each of which simultaneously sampled each of the model’s input parameters from an appropriate probabilistic distribution (normal distribution for vaccine efficacy, triangular distribution for disease incidence and costs, beta distribution for disutility). PSA was conducted to evaluate the effect of the uncertainty of input data while comparing the vaccines. PSA was completed for the base-case health outcomes and cost results comparing PHiD-CV with no vaccination and comparing PHiD-CV with PCV-13 for Peru.

### Scenario analysis

In addition to the base-case scenario, 10 scenarios of interest were also analyzed:

 1. A discount rate of 5% for costs and health effects (this higher discount rate is being recommended in some Latin American countries).

 2. No vaccine efficacy of PHiD-CV against NTHi ID, and against NTHi in ID & AOM.

 3. Costs from a social perspective (productivity losses were included, considering average annual salary for parents, employment rate, and average time lost from work for working parents of sick children).

 4. Herd effect (evaluated as a fixed incidence reduction of IPD of 15.4% and 29.0% among children <5 years and ≥5 years, respectively, based on the experience reported by CDC in the USA [50,51]). The net indirect effect on IPD was not included in base-case analysis for vaccine comparison since the potential difference in herd protection induced by these vaccines is not known. The inclusion or exclusion of equal (not differential) herd effect for all vaccines will not impact on the results because the incremental differences between them would be the same with or without inclusion of the indirect effect.

 5. No cross protection to pneumococcal type 6A in ID.

 6. No cross protection to pneumococcal type 19A in ID.

 7. A 3 + 1 vaccination scheme (at 2, 4, and 6 months of age with a booster dose at 12 months). The modeling of vaccine efficacies for this schedule has been explained previously [11].

 8. Similar vaccine efficacies of PHiD-CV and PCV-13 against IPD.

 9. No efficacy of PCV-13 against serotype 3 IPD.

 10. Equal vaccine cost per dose for all vaccines (US$ 14.24), to determine costs differences generated by vaccines at equal price per dose.

## Results

### ID, CAP, and AOM burden in Peru without vaccination

Table 2 presents the modeled health burden of pneumococcal and NTHi disease during the first 10 years of life in Peru. Although IPD is considered to generate significant mortality, in reality, pneumonia is responsible for the majority (93%) of pneumococcal/NTHi deaths in Peru (Table 2). The lifetime burden associated with pneumonia was the highest, accounting for 90% of all QALYs lost (data not shown). IPD (meningitis and bacteremia) accounted for 9% of all QALYs lost, and AOM, a mild but frequent condition, only accounted for 1% of all QALYs lost. Regarding the lifetime costs to the health system, pneumonia was responsible for 63% of all direct medical costs, AOM for 25%, and ID for 12% (data not shown).

**Table 2:** Predicted health impact of PCVs in Peru during the first 10 years of life (undiscounted data)

	**No vaccination**		**PCV-7**			**PCV-13**			**PHiD-CV**		
	**Cases**^**a**^	**Rate**^**b**^	**Cases**^**a**^	**Rate**^**b**^	**Averted (%)**	**Cases**^**a**^	**Rate**^**b**^	**Averted (%)**	**Cases**^**a**^	**Rate**^**b**^	**Averted (%)**
Cases											
Pneumococcal meningitis	177	35.4	142	28.4	19.8	129	25.8	27.1	131	26.2	26.0
NTHi meningitis	16	3.2	16	3.2	0.0	16	3.2	0.0	14	2.8	12.5
Pneumococcal bacteremia	495	98.9	350	69.9	29.3	295	58.9	40.4	303	60.5	38.8
NTHi bacteremia	30	6.0	30	6.0	0.0	30	6.0	0.0	25	5.0	16.7
Hospitalized pneumonia	37,110	7,412	32,585	6,508	12.2	31,930	6,377	14.0	31,930	6,377	14.0
Ambulatory pneumonia	39,352	7,859	38,404	7,670	2.4	38,268	7,643	2.8	38,267	7,643	2.8
AOM	306,989	61,312	297,535	59,424	3.1	286,598	57,239	6.6	270,573	54,039	11.9
Myringotomies	7,193	1,437	6,315	1,261	12.2	5,325	1,064	26.0	3,838	767	46.6
Deaths											
Pneumococcal meningitis	9.7	1.9	7.2	1.4	26.2	6.2	1.2	36.0	6.4	1.3	34.5
NTHi meningitis	1.6	0.3	1.6	0.3	0.0	1.6	0.3	0.0	1.4	0.3	10.9
Pneumococcal bacteremia	76.2	15.2	53.4	10.7	30.0	44.7	8.9	41.3	46.1	9.2	39.6
NTHi bacteremia	3.0	0.6	3.0	0.6	0.0	3.0	0.6	0.0	2.5	0.5	17.2
Pneumonias	1,212.0	242.1	1,078.4	215.4	11.0	1,059.0	211.5	12.6	1,059.0	211.5	12.6
Total	1,302.5	260.1	1,143.5	228.4	12.2	1,114.6	222.6	14.4	1,115.4	222.8	14.4

^a^Cases and deaths during the first 10 years of life.

^b^Rates expressed per 100,000 individuals.

AOM, acute otitis media; NTHi, non-typeable *Haemophilus influenzae*; PCV-7, 7-valent pneumococcal conjugate vaccine; PCV-13, 13-valent pneumococcal conjugate vaccine; PHiD-CV, 10-valent pneumococcal non-typeable Haemophilus influenzae protein D conjugate vaccine.

### Effects of vaccines on ID, CAP, and AOM

Vaccination with PCV-7, PCV-13, or PHiD-CV is estimated to reduce ID cases (pneumococcal and NTHi meningitis and bacteremia), by 25%, 35%, and 34%, respectively (Table 2). Vaccination with any of the three vaccines is expected to reduce hospitalized and ambulatory pneumonia cases by around 12-14% and 2-3%, respectively (Table 2). The burden associated with AOM is expected to decrease by 3.1%, 6.6%, and 11.9% for PCV-7, PCV-13, and PHiD-CV, respectively, with reductions in myringotomies of 12.2%, 26.0%, and 46.6%, respectively (Table 2). Since vaccine efficacies against *S. pneumoniae* for AOM were similar for all three vaccines, these differences are mostly explained by the difference in efficacy against NTHi shown by PHiD-CV.

### Effects of vaccine on deaths and QALYs gained

The results of this modeling exercise suggest that vaccination is expected to avert approximately 28.1% (PCV-7), 38.6% (PCV-13), and 37.8% (PHiD-CV) of deaths attributed to ID (pneumococcal and NTHi meningitis and bacteremia) (Table 2). This would fall slightly to 37.0% for PHiD-CV if efficacy against NTHi is not considered. PCV-13 and PHiD-CV are expected to avert 12.6% of deaths associated with pneumonia, while PCV-7 is estimated to avert 11.0% (Table 2). Overall, PCV-13 is expected to prevent 0.8 more deaths than PHiD-CV (the model estimated that 0.7 additional deaths would occur with PHiD-CV if efficacy to NTHi ID is not considered), with both vaccinations saving nearly 190 lives during the first 10 years of life of the cohort.

The greatest QALYs (morbidity and mortality) gained by vaccination in Peru is expected to be associated with pneumonia (78-82%) (Table 3). QALYs gained due to the prevention of ID and AOM account for 18-21% and 0.5-1.6% of QALYs gained, respectively (Table 3). Over the life-time of the cohort, PCV-13 is expected to avert 53.6 more QALYs (morbidity and mortality) associated with ID than PHiD-CV, but the latter is expected to avert 87.0 more QALYs (morbidity) associated with AOM than PCV-13 (undiscounted data). Overall, total QALYs gained are 2,004.2 and 2,037.6 for PCV-13 and PHiD-CV, respectively, compared with PCV-7 (Table 3).

**Table 3:** Health results of PCVs for Peru over the life-time of the cohort

		**QALYs/LYs gained**^**a**^		
		**PCV-7**	**PCV-13**	**PHiD-CV**
Pneumococcal ID	QALYs - morbidity	259.3	356.1	340.2
	QALYs - mortality	1,613.0	2,223.0	2,129.0
NTHi ID	QALYs - morbidity	0	0	12.3
	QALYs - mortality	0	0	44.0
Pneumonia	QALYs - morbidity	41.5	47.4	47.4
	QALYs - mortality	8,526.0	9,759.0	9,759.0
AOM	QALYs - morbidity	50.5	109.0	196.0
Total QALYs gained		10,490.3	12,494.5	12,527.9
Total LYs gained		11,847.0	14,000.0	13,942.0

^a^Undiscounted data.

AOM, acute otitis media; ID, invasive disease; LY, life year; NTHi, non-typeable *Haemophilus influenzae*; PCV, pneumococcal conjugate vaccine; PCV-7, 7-valent pneumococcal conjugate vaccine; PCV-13, 13-valent pneumococcal conjugate vaccine; PHiD-CV, 10-valent pneumococcal non-typeable Haemophilus influenzae protein D conjugate vaccine; QALY, quality-adjusted life year.

PCV-13 is predicted to generate 14,000 LYs gained, PHiD-CV is predicted to generate 13,942 LYs gained, and PCV-7 is predicted to generate 11,847 LYs gained, with most of them linked to pneumonia for the three vaccines (Table 3).

### Treatment costs averted, vaccine costs, and net costs

Using undiscounted data, PCV-7 vaccination is expected to save US$ 1.4 million in direct medical costs over the life-time of the cohort, PCV-13, US$ 1.9 million, and PHiD-CV, US$ 2.3 million (Table 4). This is largely attributable to cost savings for pneumonia (US$ 1.0-1.2 million; 51-73% of total) and AOM (US$ 0.2-0.9 million; 17-41% of total) (Table 4). Not surprisingly, PHiD-CV is predicted to avert more AOM costs than PCV-7 or PCV-13 (US$ 935,558 vs US$ 240,962 and US$ 521,999, respectively) due to its effect on NTHi AOM (Table 4). Although the studied vaccines present different coverage against the pneumococcal types prevalent in ID in Peru, they do not show large differences in the predicted economic burden averted (US$ 0.1-0.2 million) (Table 4).

**Table 4:** Economic results of PCVs for Peru

	**PCV-7**		**PCV-13**		**PHiD-CV**	
	**Costs averted**^**a**^	**%**	**Costs averted**^**a**^	**%**	**Costs averted**^**a**^	**%**
All ID	138,603	10.0	190,893	10.2	188,650	8.3
Meningitis (all)	19,386	1.4	26,641	1.4	26,392	1.2
Meningitis sequelae (all)	80,789	5.8	110,947	5.9	109,835	4.8
Bacteremia (all)	38,428	2.8	53,305	2.9	52,423	2.3
Pneumonia	1,008,424	72.7	1,153,782	61.8	1,153,845	50.7
All AOM	240,962	17.4	521,999	28.0	935,558	41.1
Total direct medical costs	1,387,989	100	1,866,674	100	2,278,053	100
Vaccination cost	−23,727,362		−25,487,635		−22,393,203	
Net total cost	−22,339,373		−23,620,961		−20,115,150	

^a^Undiscounted costs in 2009 US$.

A negative value indicated costs incurred.

AOM, acute otitis media; ID, invasive disease; PCV-7, 7-valent pneumococcal conjugate vaccine; PCV-13, 13-valent pneumococcal conjugate vaccine; PHiD-CV, 10-valent pneumococcal non-typeable *Haemophilus influenzae* protein D conjugate vaccine.

Considering the PAHO Revolving Fund Program cost for each vaccine during 2012, the cost of the vaccination program would be US$ 3.1 million lower for PHiD-CV than for PCV-13 (US$ 22.4 million vs US$ 25.5 million) (Table 4).

The predicted net total cost of a PCV vaccination program (considering direct medical cost savings and vaccination costs) is predicted to be lowest for PHiD-CV in Peru (US$ 20.1 million vs US$ 22.3 million for PCV-7 and US$ 23.6 million for PCV-13; Table 4).

### Cost utility and cost-effectiveness analysis

Tables 5 and 6 present the cost utility and cost-effectiveness analysis expressed as ICERs in US$ per QALY (Table 5) or LY (Table 6) saved versus no vaccination and comparing the three vaccines. PCV-13 was estimated to generate 719 more QALYs gained than PCV-7 at an additional investment of US$ 1.3 million, making it a cost-effective intervention compared to PCV-7 for Peru (Table 5). PHiD-CV was estimated to generate 769 more QALYs gained than PCV-7 with a reduced investment (−US$ 2.1 million); in addition, PHiD-CV was estimated to generate 50 more QALYs gained than PCV-13 with a reduced investment (−US$ 3.4 million) (Table 5). The negative ICER for PHiD-CV in Table 5 shows that PHiD-CV is predicted to be cost saving per QALY gained compared to PCV-7 and PCV-13 for Peru when quality of life is considered.

**Table 5:** Cost-effectiveness of PCVs (per QALY gained), in increasing order of QALYs

	**QALYs**	**Cost**^**a**^	**Differences vs no vaccine**			**Incremental differences vs PCV-7 scenario**		
			**QALYs**	**Cost**^**a**^	**ICER**	**QALYs**	**Cost**^**a**^	**ICER**
No vaccine	11,586,536	23,570,416	-	-	-	-	-	-
PCV-7	11,590,206	45,641,050	3,670	22,070,634	6,014	-	-	-
PCV-13	11,590,925	46,952,004	4,389	23,381,588	5,327	719	1,310,954	Dominated
PHiD-CV	11,590,975	43,544,180	4,439	19,973,764	4,500	50	−3,407,824	−2,727

^a^Costs in 2009 US$ (discounted data).

ICER, incremental cost-effectiveness ratio; PCV, pneumococcal conjugate vaccine; PCV-7, 7-valent pneumococcal conjugate vaccine; PCV-13, 13-valent pneumococcal conjugate vaccine; PHiD-CV, 10-valent pneumococcal non-typeable Haemophilus influenzae protein D conjugate vaccine; QALY, quality-adjusted life year.

**Table 6:** Cost-effectiveness of PCVs (per LY gained) in increasing order of LYs

	**LYs**	**Cost**^**a**^	**Difference vs no vaccine**			**Incremental differences vs PCV-7 scenario**		
			**LYs**	**Cost**^**a**^	**ICER**	**LYs**	**Cost**^**a**^	**ICER**
No vaccine	12,988,553	23,570,416	-	-	-	-	-	-
PCV-7	12,992,507	45,641,050	3,954	22,070,634	5,582	-	-	-
PHiD-CV	12,993,206	43,544,180	4,653	19,973,764	4,293	699	−2,096,870	−3,000
PCV-13	12,993,226	46,952,004	4,673	23,381,588	5,004	20	3,407,824	170,391

^a^Costs in 2009 US$ (discounted data).

ICER, incremental cost-effectiveness ratio; LY, life year; PCV, pneumococcal conjugate vaccine; PCV-7, 7-valent pneumococcal conjugate vaccine; PCV-13, 13-valent pneumococcal conjugate vaccine; PHiD-CV, 10-valent pneumococcal non-typeable Haemophilus influenzae protein D conjugate vaccine.

Table 6 shows that PHiD-CV is also predicted to be the most cost-effective vaccine when quality of life is not considered (US$ 4,293 per LY). Furthermore, PHiD-CV is predicted to be cost saving compared to PCV-7, generating 699 additional LYs at a reduced investment (−US$ 2.1 million) (Table 6). Although PCV-13 is predicted to save 20 more LYs than PHiD-CV, at an incremental investment of US$ 3.4 million (compared to PHiD-CV), the ICER of PCV-13 versus PHiD-CV is US$ 170,391 per LY saved (greater than the 3 GDP per capita threshold for Peru) (Table 6).

Overall, all three vaccines are predicted to be cost-effective for Peru compared with no vaccination, using the GDP/Capita threshold developed by the WHO [48,49]. However, PHiD-CV is predicted to be the most cost-effective vaccine, whether costs per QALY or costs per LY are considered.

### Sensitivity analysis

**Figure 1:**
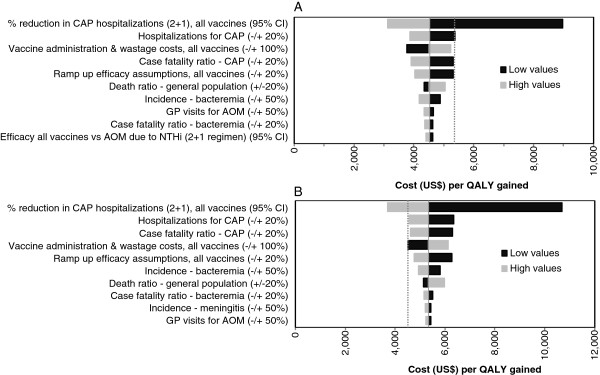
**One-way sensitivity analysis of ICERs. A**: PHiD-CV vs no vaccine; **B**: PCV-13 vs no vaccine. Dashed line in **A** shows the ICER for PCV-13 vaccine and in **B** shows the ICER PHiD-CV vaccine. AOM, acute otitis media; CAP, community acquired pneumonia; CI, confidence interval; GP, general practitioner; ICER, incremental cost-effectiveness ratio; PCV-13, 13-valent pneumococcal conjugate vaccine; PHiD-CV, 10-valent pneumococcal non-typeable Haemophilus influenzae protein D conjugate vaccine.

Figure 1 displays the results of the one-way sensitivity analysis to determine the effects of uncertainty in model input parameters on the cost-effectiveness results of the model. The analysis of the ICERs of PHiD-CV versus no vaccination (Figure 1A) shows that only the vaccine efficacy against hospitalized pneumonias may significantly modify its value. Nevertheless, the ICER in the worst scenario (with lower efficacy to hospitalized pneumonias), is not greater than the 3 GDP per capita threshold of cost-effectiveness. Similar results are observed for PCV-13 (Figure 1B).

**Figure 2:**
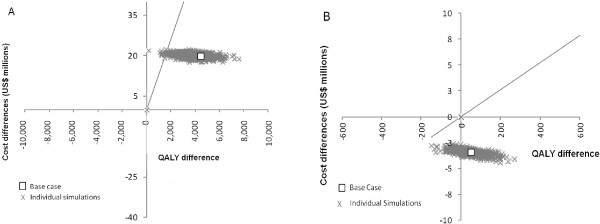
**Probabilistic sensitivity analysis. A**: ICER of PHiD-CV vs no vaccine; **B**: ICER of PHiD-CV vs PCV-13. The diagonal grey line indicates the cost-effectiveness threshold. ICER, incremental cost-effectiveness ratio; PCV-13, 13-valent pneumococcal conjugate vaccine; PHiD-CV, 10-valent pneumococcal non-typeable Haemophilus influenzae protein D conjugate vaccine; QALY, quality-adjusted life year.

The PSA on the ICER of PHiD-CV versus no vaccination (Figure 2A) in the base-case scenario confirms that PHiD-CV is a cost-effective intervention in 99.9% of simulations. In addition, Figure 2B, which compares the ICERs of PHiD-CV versus PCV-13, shows that PHiD-CV generates more QALYs gained at a reduced investment in 84% of the simulations and less QALYs gained at a reduced investment in 16% of the simulations.

### Scenario analysis

**Figure 3:**
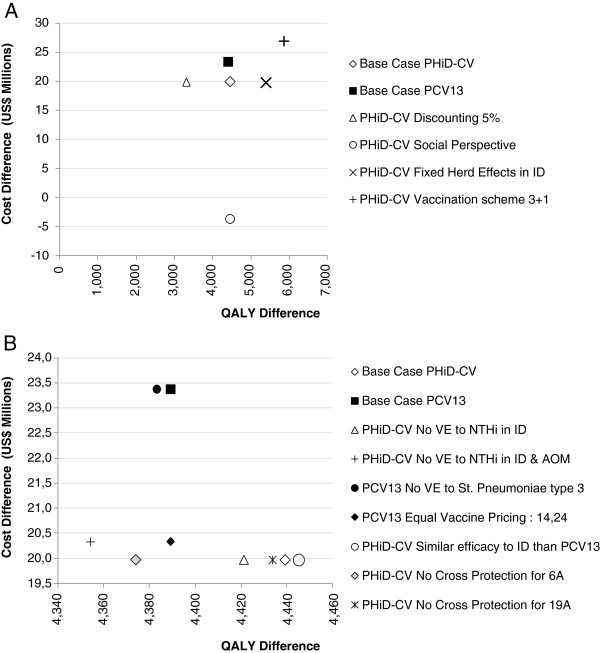
**Scenario analysis comparing PHiD-CV and PCV-13 vs no vaccine. A**. contains scenarios generating greater variations on health effects and costs. **B**. contains scenarios generation smaller variations on health effects and costs. ID, invasive disease; NTHi, non-typeable *Haemophilus influenzae*; PCV-13, 13-valent pneumococcal conjugate vaccine; PHiD-CV, 10-valent pneumococcal non-typeable Haemophilus influenzae protein D conjugate vaccine.

Figure 3 shows the results of the different scenarios of interest analyzed. Using a discount rate of 5% reduced (by 26%) the estimated number of QALYs gained with PHiD-CV. The largest difference was found when considering the indirect costs using a social perspective. This made PHiD-CV a cost-saving intervention compared to no vaccination (Figure 3A). When herd effects were considered, a slightly greater effect on QALYs gained was seen (21%). A 3 + 1 schedule increased the number of QALYs gained (32%) and also the investment required (35%) (Figure 3A). In the scenario with equal pricing of PHiD-CV and PCV-13, the investment needed for PCV-13 is reduced by 13%, but it is still higher than the investment required for PHiD-CV, due to AOM cost offsets. The scenario with no efficacy against NTHi in ID and AOM shows a decrease in the QALYs gained by PHiD-CV with minor changes in costs. Finally, the scenarios with no efficacy for PHiD-CV against NTHi ID, no efficacy for PCV-13 against type 3 IPD, no cross protection to 6A and 19A, or with similar efficacies to IPD between vaccines do not present significant changes compared to the base-case analysis.

## Discussion

The adoption of a new vaccination program requires evidence-based and informed policy decision-making. The aim of our project was to provide cost-utility and cost-effectiveness data on the new PCVs to help the decision-making process at the national level of Peru. Although this is purely a modeling exercise, some conclusions can be highlighted. Pneumonia was the main driver for pneumococcal disease burden in Peru. Without vaccination, it was estimated to generate 90% of all QALY lost and 63% of all direct medical costs in Peru. PHiD-CV and PCV-13 are predicted to prevent a similar number of deaths, but PHiD-CV is predicted to generate 33.4 additional QALYs at a reduced cost of US$ 3.5 million (undiscounted data). The benefits observed for PHiD-CV over PCV-13 are mainly due to the vaccine effects on AOM. PHiD-CV is predicted to generate 87.0 additional QALYs (~80% more than PCV-13) and save US$ 0.4 million (~80% more than PCV-13) associated with the prevention of AOM. Other results were similar for PHiD-CV and PCV-13. However, a PHiD-CV vaccination program would cost US$ 3.1 million less than a PCV-13 vaccination program, based on the 2012 vaccine prices of the PAHO Revolving Fund, resulting in an estimated net total cost saving for PHiD-CV versus PCV-13 of US$ 3.5 million (undiscounted data).

In addition, all vaccines are predicted to be cost-effective for Peru, and close to the highly cost-effective threshold, generating major health and economic benefits for this Latin American country. A similar result was obtained in a recent study carried out for PHiD-CV in several Latin American countries including Peru [14]. When only LYs lost were considered (i.e., no quality of life factors analyzed), both vaccines were predicted to be cost-effective for Peru, PHiD-CV more so than PCV-13, based on the additional economic burden averted and the reduced cost per dose. The net total cost required for PHiD-CV vaccination is predicted to be US$ 3.4 million less than for PCV-13. Additionally, PCV-13 is predicted to generate 20 additional LY compared to PHiD-CV at an extra cost of US$ 3.4 million, giving an ICER of US$ 170,391 per LY saved (over the cost-effectiveness threshold defined for Peru), and PHiD-CV is predicted to be cost saving compared to PCV-7 (generates 699 additional LYs at a reduced cost of US$ 2.1 million). Considering quality of life in the analysis, PHiD-CV and PCV-13 present similar health benefits related to ID and pneumonia outcomes. However, PHiD-CV shows a slightly better health profile based on its effects on AOM. In addition, PHiD-CV has a much better economic profile, based on its higher economic impact on AOM treatment and its lower price per dose in the PAHO Revolving Fund. Overall, PCV-13 is dominated by PHiD-CV because PHiD-CV generates 50 more QALYs gained at a reduced cost of US$ 3.4 million (discounted data), but it is more cost effective than PCV-7. Similar to the scenario without quality of life, PHiD-CV is predicted to be cost saving compared to PCV7 (generates 769 additional QALYs at a reduced cost of US$ 2.1 million). The PSA shows that PCV-13 is dominated by PHiD-CV in 84% of the simulations, and PHiD-CV presents a better economic profile in the other 16% of the simulations.

It is important to evaluate our results after considering several assumptions with the input data used in this model. The most important assumption is the paucity of efficacy data for the new vaccines. This limitation was minimized by using estimates of vaccine efficacy based on serotype-specific efficacies for ID demonstrated for the pneumococcal serotypes included in PCV-7 [24] and the pneumococcal serotype prevalence in ID described by the SIREVA surveillance network for Peru [21,22]. A recent study [52] on the efficacy of PHiD-CV against ID confirms our efficacy assumptions. For pneumonia, various clinical trials of different PCVs and a Cochrane review [25-31,33-35] do not show a relationship between pneumococcal type coverage and efficacy against pneumonia outcomes. Therefore, we used the same efficacy estimates against pneumonia for all three vaccines using data from a recently completed clinical trial of PHiD-CV (performed entirely in Latin American countries) to determine its efficacy against pneumonia [33-35]. Another limitation of this analysis is the quality of the epidemiological data available for Peru, which may impact on the accuracy of the assessment of the cost-effectiveness of these vaccines. We based our disease burden estimations on mortality data and other statistics reported for Peru, but also on expert opinion consultations when information was missing. Detailed explanation on these procedures were previously published [14], but we recognize this limitation and the uncertainty around these input. Published estimates of pneumococcal disease burden generated for the Latina region by two international organizations - the Sabin Vaccine Institute/Pan American Health Organization [2] and the WHO [1] - were significantly different. This is probably as a result of different literature review processes or modeling strategies, but it shows the difficulties of this task and should not be ignored when these results are analyzed. Although great efforts were taken to assess the completeness and coverage of the reporting systems evaluated and used in the present study, this is an important limitation to consider when analyzing this cost effectiveness study.

We can compare our results with other cost-effectiveness studies comparing PCVs, particularly in Latin American countries [53-55]. Muciño-Ortega et al. [53] presented an analysis of PCVs for Mexico and showed PCV-13 to be dominant over PHiD-CV and PCV-7. They showed, surprisingly, that PCV-13 (with three additional pneumococcal types) is expected to prevent three times as many deaths as PHiD-CV. This study used ecological studies to estimate vaccine efficacy against pneumonia, ignoring many clinical trials of PCVs against this specific outcome. In addition, they adjusted vaccine efficacy against pneumonia with the estimated serotype coverage for each vaccine. Finally, they did not consider the vaccine efficacy of PHiD-CV against AOM-related outcomes generated by Prymula et al. [13]; instead, they calculated an adjusted vaccine efficacy against AOM based on selected immunogenicity data comparing PHiD-CV and PCV-7. It is difficult to analyze their overall results when they prioritized the use of ecological or immunogenicity studies over clinical trials for the estimation of vaccine efficacy. Urueña et al. [54] described that either PHiD-CV or PCV-13 would be cost effective for Argentina, with ICERs of US$ 8,973/DALY and US$ 10,948/DALY, respectively. Their analysis shows better incremental cost differences for PHiD-CV (at price parity per dose) and better incremental health gains for PCV-13. Although they mentioned that serotype coverage adjustments were not assumed for vaccine efficacy against consolidated pneumonia (same assumption that we used; equal vaccine efficacy), the most significant difference in health gains between the vaccines was the differential vaccine effects observed against pneumonia. If vaccine effects on consolidated pneumonia were adjusted in line with their methods and our assumptions, both studies would be very much aligned in their conclusions. Lastly, Castañeda-Orjuela et al. [55] have described that PHiD-CV and PCV-13 would be cost-effective interventions in Colombia, with ICERs of US$ 1,837/LY and US$ 2,742/LY, respectively. This analysis also shows better incremental health gains for PCV-13, but no evaluation of quality of life was included in the base-case or any other scenario analyzed. In addition, this study also reports better incremental cost differences for PHiD-CV (compared to PCV-13), based on its better profile against AOM, although prices per dose were not totally comparable. They used US$ 14.85 for PHiD-CV (price for the PAHO 2011 RF) and US$ 16.34 for PCV-13. Although the price for PCV-13 was provided by the Ministry of Health this was the vaccine price for the PAHO 2012 RF, and was not available in 2011 for any public source. In conclusion, the latest two studies are in agreement with our conclusion for vaccine comparison, in the scenario without considering quality of life. The additional health benefits provided by PCV-13 over PHiD-CV require significant investment and the ICER is over the 1 GDP per capita threshold defined for developing countries [48,49]. Overall, the results presented in these Latin American studies [54,55] are well aligned with each other, and if inconsistencies were removed (differential vaccine effects on pneumonia for the Argentine study [54]) or more complete scenarios were evaluated (quality of life included in the Colombian study [55]), the conclusions of these two studies would be even closer to ours, highlighting the robustness of our study.

Our results could change slightly if vaccine prices at the PAHO RF were different. We analyzed the scenario with equal vaccine prices and showed that part of the cost benefits identified for PHiD-CV is reduced. Nevertheless, PHiD-CV will still generate additional QALYs gained at a reduced cost (compared to PCV-13) in this modified scenario for vaccine costs. Both PCV-13 and PHiD-CV have participated in the PAHO RF fund since 2012 and PHiD-CV consistently presented reduced cost per dose compared to PCV-13.

Although this is purely a modeling exercise, with all its caveats, the information presented is a critical tool for decision makers because it is the only way to merge different evidences in order to generate one analysis for decision-making purposes. Dissemination of this information will contribute to evidence-based decision-making about the introduction of new vaccines in Latin American countries.

## Conclusions

The results of this modeling study predict that PCVs are likely to be a cost-effective strategy to help relieve the epidemiological and economic burden associated with pediatric pneumococcal and NTHi diseases for Peru. PHiD-CV is likely to be a dominant (better health gains at a reduced net cost) intervention compared to PCV-13 or PCV-7. The most significant drivers for these results are the better health and economic profile of PHiD-CV against AOM and its reduced cost per dose available through the PAHO Revolving Fund in the LAC region.

## Abbreviations

AOM: Acute otitis media; CAP: Community acquired pneumonia; EMEA: European medicines agency; GDP: Gross domestic product; ICER: Incremental cost-effectiveness ratio; ID: Invasive disease; IPD: Invasive pneumococcal disease; LAC: Latin America and Caribbean; LY: Life years; NTHi: Non-typeable *Haemophilus influenzae*; PAHO: Pan American health organization; PCV-7: 7-valent pneumococcal conjugate vaccine; PCV-13: 13-valent pneumococcal conjugate vaccine; PHiD-CV: 10-valent pneumococcal non-typeable *Haemophilus influenzae* protein D conjugate vaccine; PSA: Probabilistic sensitivity analyses; QALY: Quality-adjusted life year; WHO: World health organization.

## Competing interests

JAG, AANR, MMCA, and OT are employees of GlaxoSmithKline. JCT has received travel, accommodation, and meeting expenses to a Latin-American congress related to vaccines, sponsored by Pfizer; and payment for speaker services in Perú, sponsored by GlaxoSmithKline. This study was sponsored by GlaxoSmithKline Vaccines. Study sponsors were involved in the study design, in the analysis and interpretation of data, in the writing of the manuscript, and in the decision to submit the manuscript for publication. GlaxoSmithKline financed the study and is paying the article-processing charge.

## Authors’ contributions

JAG, JCT, AANR, and MMCA participated in acquisition, analysis and interpretation of data and in the overall modeling exercise. JCT made critical decisions as expert when no reference was found in the literature. JAG wrote the first draft. OT was involved in the model development and analysis of data. All authors performed critical revisions of drafts and read and approved the final manuscript.

## Pre-publication history

The pre-publication history for this paper can be accessed here:

http://www.biomedcentral.com/1471-2458/13/1025/prepub

## Supplementary Material

Additional file 1 Additional inputs used to calibrate the Markov model for Peru with its corresponding references. Click here for file
