# Supplementary material for: Cost-effectiveness and cost utility analysis of three pneumococcal conjugate vaccines in children of Peru
Source: BMC Public Health. 2013 Oct 30;13:1025. doi: 10.1186/1471-2458-13-1025 (PMC4228443; doi:10.1186/1471-2458-13-1025)
Supplement: Additional file 1 — Additional inputs used to calibrate the Markov model for Peru with its corresponding references. [file 1471-2458-13-1025-S1.pdf]

Supplementary information

Calculation of NTHi frequency on Invasive Disease

Based on data from the paper by Gabastou et al. [1]:

- 2,782 *Haemophilus influenzae* strains were analyzed
- 24.7% of all *H. influenzae* strains collected during 2000-2005 were non-typeable *H. influenzae* (NTHi)
- The percentage of NTHi increased with time (Figure 1A of [1]); therefore, in 2007, it is estimated that 38% of all *H. influenzae* strains were NTHi (Figure).

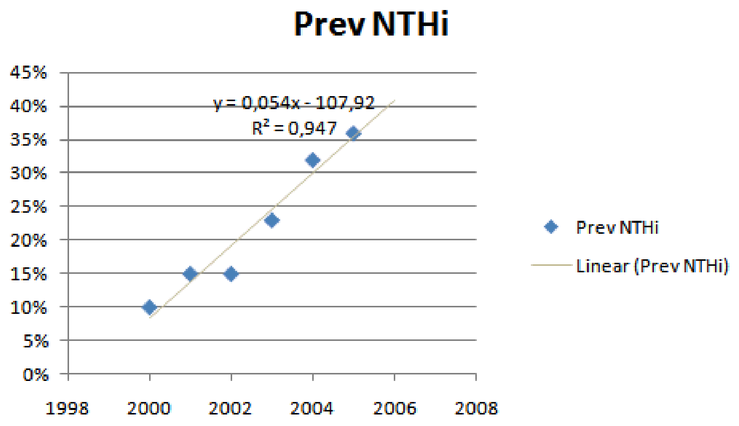

- The estimated number of NTHi strains identified in 2007 was therefore 0.38 × 2,782 = 1,057
- 1,573 *H. influenzae* strains were from meningitis (estimated NTHi = 0.38 × 1,573 = 598) and 602 *H. influenzae* strains were from sepsis/bacteremia (estimated NTHi = 0.38 × 602 = 229) (from Table 6 of [1])
- 6,753 cases of meningitis were due to *S. pneumoniae* (from Table 2 of [1]) and 3,783 cases of sepsis/bacteremia were due to *S. pneumoniae* (from Table 2 of [1])
- Therefore the ratio of *S. pneumoniae* to NTHi meningitis was assumed to be 11:1 and *S. pneumoniae* to NTHi bacteremia, 17:1.

Supplementary Table 1 Pneumococcal meningitis epidemiology by age group

Age group	Annual incidence (per 100,000 inhabitants)[a]	Fatality (%)[b]	Cases with neurological sequelae (%)[b]	Cases with hypoacusia (%)[b]
<1 year	5.1	20.0	19.4	3.4
1 year	1.4	10.0	19.4	3.4
2 years	1.4	10.0	19.4	3.4
3 years	1.9	5.0	19.4	3.4
4 years	1.9	5.0	19.4	3.4
5-9 years	4.9	2.0	19.4	3.4
10-14 years	1.0	2.0	19.4	3.4
15-19 years	4.8	2.0	18.2	4.4
20-24 years	1.8	9.3	18.2	4.4
25-29 years	1.2	9.3	18.2	4.4
30-34 years	1.1	9.3	18.2	4.4
35-39 years	2.4	9.3	18.2	4.4
40-44 years	2.4	9.3	18.2	4.4
45-49 years	2.8	12.5	18.2	4.4
50-54 years	3.8	12.5	18.2	4.4
55-59 years	3.0	12.5	18.2	4.4
60-64 years	3.0	12.5	18.2	4.4
65-69 years	2.5	25.0	18.2	4.4
70-74 years	1.9	25.0	18.2	4.4
75-79 years	2.5	25.0	18.2	4.4
80-84 years	6.7	25.0	18.2	4.4
85-89 years	6.7	25.0	18.2	4.4
≥ 90 years	6.7	25.0	18.2	4.4

[a]Based on mortality from Oficina General de Estadística e Informática (OGEI) 2006 [2] divided by the hospital fatality. The percentage of cases attributable to pneumococcus were based on Delphi panel

results from Peru (for pediatric patients) or average of Delphi panels from Chile, Brazil, Mexico and Colombia (for adults).

[b]Based on Delphi panel results from Peru (for pediatric ages) or average of Delphi panels results from Chile, Brazil, Mexico and Colombia (for adults).

Supplementary Table 2 Epidemiology of pneumococcal bacteremia by age group

Age group	Annual incidence (per 100,000 inhabitants)[a]	Fatality (%)[b]
<1 year	54.2	20.0
1 year	9.7	15.0
2 years	9.7	15.0
3 years	7.3	10.0
4 years	7.3	10.0
5-9 years	2.5	2.0
10-14 years	2.5	2.0
15-19 years	2.5	2.0
20-24 years	4.5	11.9
25-29 years	4.0	11.9
30-34 years	4.8	11.9
35-39 years	5.5	11.9
40-44 years	9.2	11.9
45-49 years	8.6	17.5
50-54 years	12.8	17.5
55-59 years	19.8	17.5
60-64 years	33.0	17.5
65-69 years	54.7	28.8
70-74 years	86.1	28.8
75-79 years	144.8	28.8
80-84 years	475.3	28.8
85-89 years	475.3	28.8
≥90 years	475.3	28.8

[a]Based on mortality from Oficina General de Estadística e Informática (OGEI) 2006 [2] divided by the hospital fatality. The percentage of cases attributable to pneumococcus was based on Peru Delphi panel results (for the pediatrics ages) or average of Delphi panels results from Chile, Brazil, Mexico and Colombia (for adults).

[b]Based on the Peru Delphi results (for the pediatrics ages) or average of Delphi panels results from Chile, Brazil, Mexico and Colombia (for adults).

Supplementary Table 3 Pneumonia epidemiology from all causes by age group

Age group	Annual hospitalization rate (per 100,000 inhabitants)[a]	Fatality (%)[b]	Annual rate of outpatient visits (per 100,000 inhabitants)[c]
<1 year	2,130	7	236
1 year	1,034	1	258
2 years	1,034	1	258
3 years	1,034	1	1,034
4 years	1034	1	1,034
5-9 years	262	1	1,050
10-14 years	196	1	787
15-19 years	22	15	89
20-24 years	24	16	109
25-29 years	31	16	142
30-34 years	30	17	136
35-39 years	36	17	165
40-44 years	56	19	254
45-49 years	70	22	135
50-54 years	83	23	159
55-59 years	156	23	298
60-64 years	233	25	446
65-69 years	262	48	174
70-74 years	442	52	294
75-79 years	820	52	546
80-84 years	2,391	65	1,594
85-89 years	2,391	65	1,594
≥90 years	2,391	65	1,594

[a]Based on mortality from Oficina General de Estadística e Informática (OGEI) 2006 [2], divided by the hospital fatality rate assuming that only 46% of deaths occur in hospital [3].

[b]Based on fatalities reported by studies from Brazil, Chile and Mexico [4, 5] and a personal communication from División de Planificación Sanitaria, Departamento de Estadísticas e Información de Salud, Ministerio de Salud de Chile, April 2009.

[3]To estimate the number of outpatient cases we used the annual hospitalization rate per 100,000 inhabitants and the percentage of pneumonia cases requiring hospitalization (based on Delphi panel from Peru [for pediatric ages] or average from Delphi panels from Chile, Brazil, Mexico and Colombia [for adults]).

Supplementary Table 4 Epidemiology of acute otitis media by any cause by age group

Age group	Annual outpatients cases (per 100,000 inhabitants)[a]	Annual myringotomies (per 100,000 inhabitants)[b]	Annual cases with sequelae (per 100,000 inhabitants)[c]
<1 year	8943.5	89.4	1207.4
1 year	8136.5	366.2	691.6
2 years	7151.6	321.8	607.9
3 years	7950.7	238.5	318.0
4 years	7523.4	225.7	300.9
5-9 years	4626.9	46.3	69.4
10-14 years	4626.9	46.3	69.4
15-19 years	1133.7	11.3	17.0
20-24 years	969.3	41.2	50.9
25-29 years	908.5	38.6	47.7
30-34 years	908.5	38.6	47.7
35-39 years	908.5	38.6	47.7
40-44 years	908.5	38.6	47.7
45-49 years	841.5	25.2	69.4
50-54 years	841.5	25.2	69.4
55-59 years	841.5	25.2	69.4
60-64 years	846.9	25.4	69.9
65-69 years	709.2	39.0	94.9
70-74 years	709.2	39.0	94.9
75-79 years	709.2	39.0	94.9
80-84 years	709.2	39.0	94.9
85-89 years	709.2	39.0	94.9
≥90 years	709.2	38.6	94.9

[a]Based on the average of incidences reported by studies from Brazil, Chile and Mexico [6-8].

[b]Calculated based on the incidence of outpatient cases and the percentage of cases that would require myringotomy (based on Delphi panel from Peru [for pediatric patients] or average from Delphi panels from Chile, Brazil, Mexico and Colombia [for adults]).

[c]Calculated based on the incidence of outpatient cases and the percentage of cases with sequelae (based on Delphi panel from Peru [for pediatric ages] or Delphi panels average from Chile, Brazil, Mexico and Colombia [for adults]).

Supplementary Table 5 *S. pneumoniae* serotype distribution[a]

Age group	Invasive disease (%)			
	**0-2 years**	**2-5 years**	**5-10 years**	**>10 years**
1	1.7	1.7	3.5	3.5
3	0.6	0.6	0.0	0.0
4	1.1	1.1	0.0	0.0
5	8.1	8.1	6.8	6.8
6A	7.5	7.5	3.4	3.4
6B	8.1	8.1	3.4	3.4
7F	0.0	0.0	0.0	0.0
9V	1.7	1.7	3.5	3.5
14	40.3	40.3	26.5	26.5
18C	1.2	1.2	13.7	13.7
19A	1.7	1.7	5.1	5.1
19F	3.4	3.4	14.5	14.5
23F	2.3	2.3	10.3	10.3
Others	22.3	22.3	9.4	9.4

[a]Based on SIREVA II [9, 10].

Supplementary Table 6 Costs included in the model[a]

	**Public scenario costs**		**EsSalud scenario costs**		**Private scenario costs**		**Weighted scenario costs**	
	**Children**	**Adults**	**Children**	**Adults**	**Children**	**Adults**	**Children**	**Adults**
Average cost per acute episode								
Pneumonia (hospitalized patients)	278.84	417.94	1,395.61	3,331.54	2,538.11	3,726.26	605.95	926.17
Pneumonia (outpatients)	68.17	112.15	151.63	208.19	293.89	414.28	95.93	146.56
Myringotomy	41.92	45.88	147.60	160.81	300.00	326.91	75.46	82.38
Acute otitis media (outpatients)	41.06	38.43	109.08	99.86	202.81	185.32	62.38	57.73
Meningitis (hospitalized patients)	633.52	627.58	4,033.98	3,827.41	6,079.50	5,870.59	1,545.21	1,492.42
Bacteremia (hospitalized patients)	309.09	446.89	1,938.00	3,176.69	3,400.50	5,037.69	774.28	1,191.77
Average cost per sequelae								
Neurologic sequel due to meningitis (non-hypoacusia)	620.38	166.63	1,842.00	494.76	2,657.82	693.88	952.15	254.57
Hypoacusia	40.27	150.74	87.00	336.02	138.61	492.32	54.16	202.97

[a]All costs [11-14] were measured in 2009 Nuevos Soles as shown in present table. These costs were converted to 2009 US$ based on the exchange rate of US$1 = 2.78 Nuevos Soles, for the analysis.

Supplementary Table 7 Yearly utility decrements

	Decrement	Source
Short-term disutilities associated with acute pathologies[a]		
Pneumonia (hospitalized)	0.008	Assumed to be the same as for hospitalized bacteremia [15]
Pneumonia (ambulatory)	0.006	[15]
AOM (ambulatory)	0.005	[16]
AOM with myringotomy	0.005	Assumed the same as for AOM without myringotomy
Pneumococcal meningitis	0.023	[15]
Pneumococcal bacteremia	0.008	[15]
Long-term disutilities associated with sequelae[b]		
Hipoacusia due to AOM	0.090	[17]
Neurologic sequelae due to meningitis	0.400	[18]
Hipoacusia due to meningitis	0.200	[18]

[a]Applied to current year without discount.

[b]Applied to current and subsequent years with discount.

AOM, acute otitis media.

Supplementary Table 8 Parameters included in the sensitivity analysis comparing PHiD-CV and PCV-13 versus no vaccination

Variable	Base case	Distribution Type	Range of sensitivity analysis
Pneumonia incidence	Age-specific data (see Table 3)	Triangular	-/+20% for hospitalizations<br>-/+50% for ambulatory cases
Pneumonia case fatality ratio	Age-specific data (see Table 3)	Triangular	-/+20%
AOM incidence	Age-specific data (see Table 4)	Triangular	-/+20% for myringotomies<br>-/+50% for total cases
AOM etiology	Pneumo AOM: 35.9%<br>NTHi AOM: 32.3%<br>Pneumos covered by<br>PHiD-CV: 76.2%;<br>PCV-13: 89.5%.<br>PCV-7: 69.8%<br>[24, 25]	Triangular	-/+20% for Sp cases<br>-/+20% for NTHi cases
Meningitis incidence	Age-specific data (See Table 1)	Triangular	-/+50%
Meningitis case fatality ratio	Age-specific data (See Table 1)	Triangular	-/+20%
Meningitis risk of sequelae	Age-specific data (See Table 1)	Triangular	-/+20%
Bacteremia incidence	Age-specific data (See Table 2)	Triangular	-/+50%
Bacteremia case fatality ratio	Age-specific data (See Table 2)	Triangular	-/+20%

ID etiology (Sp serotype distribution)	Age-specific data (See Table 5)	Dirichlet	95% CI
ID etiology (Sp in ≥10 years)	Age-specific data (See Table 5)	Dirichlet	-/+20%
Effectiveness in reducing hospitalizations for pneumonia	23.4% [19-21]	Lognormal	95% CI
Effectiveness in reducing ambulatory pneumonia	7.3% [19-21]	Lognormal	95% CI
Efficacy in preventing AOM according to type of pathogen	57.6% for SpC; -33% for SpNC; 35.3% for NTHi [22, 23]	Lognormal	95% CI
Efficacy in preventing NTHi associated ID	Based on Prymula et al 2006 [22]	Triangular	-/+ 20%
Efficacy in preventing PID	Based on efficacy by serotype for PCV-7 [26]	Lognormal	95% CI for each serotype
Costs of administration and vaccine wastage	US$1 with 10% wastage	Triangular	-/+100%
Direct cost of treatment of acute events	Disease-specific data (See Table 6)	Triangular	-/+20%
Annual cost of long-term treatment of sequelae	Data specific for each condition (see Table 6)	Triangular	-/+20%
Disutilities associated with AOM (with and without myringotomy)	Data specific for each condition (see Table 7)	Beta	95% CI
Disutilities associated with acute diseases except AOM[a]	Data specific for each condition (see Table 7)	Triangular	95% CI
Disutilities associated with	Data specific for each	Normal for	95% CI

chronic conditions (sequelae)[b]	condition (see Table 7)	hearing loss Triangular for Sequelae	
Death ratio - general population	Age-specific data [27]	Triangular	-/+ 20%
Ramp up efficacy assumptions (all vaccines)	Increase to maximum (published) value with number of doses	Triangular for dose 0-2<br>Lognormal for dose 3	-/+ 20%<br>IC95%

[a]Per episode. [b]Per year. AOM, acute otitis media; CI, confidence interval; ID, invasive disease; NTHi, non-typeable *Haemophilus influenzae*; Sp, *Streptococcus pneumoniae*; SpC: *S. pneumoniae* serotypes covered by vaccine; SpNC: *S. pneumoniae* serotypes not covered by vaccine.

Pneumoccocal Vaccine and Modelling experts collaborating with model development

Advisory Board: 7–8 March 2008, Montreal, Canada

Participants

Advisory board members – Prof Steven Black (Chairman), Prof Philippe De Wals (Principal Model Developer), Dr Arto Palmu, Prof Stephen Pelton, Prof David Scheifele, Prof Catherine Weil-Olivier

Observers – Dr Lonny Erickson, Dr Geneviève Petit, Dr Beatrice Poirier

GlaxoSmithKline Biologicals – Dr William Hausdorff, Mrs Suzanne Laplante, Mr Pierrick Rollet, Mr Jean-Bernard Simeon, Dr Baudouin Standaert, Dr Tom Taylor

Fishawack Communications – Mr Tim Mustill, Dr Joanne Cook, Miss Gemma Hayden

Advisory board: 19 October 2007, Dublin, Ireland

Participants

Advisory board members – Prof Philippe De Wals (Chairman), Dr Pierre-Yves Boëlle,
Dr Ray Borrow, Prof Jim Butler, Prof Paolo Castiglia, Professor Gabriela Echániz-Aviles,
Prof Stephen Pelton, Dr Geneviève Petit, Dr Mark Van der Linden

GlaxoSmithKline Biologicals – Ms Rachel Emerson, Dr William Hausdorff, Mrs Suzanne Laplante, Mr Jean-Bernard Simeon, Dr Tom Taylor, Dr Robert Welte, Dr Thierry Van Effelterre

Fishawack Communications – Mr Tim Mustill, Dr Diane Sutherland, Ms Gemma Hayden, Mr Neil Whitely

References

1. Gabastou JM, Agudelo CI, Brandileone MC, Castaneda E, de Lemos AP, Di Fabio JL, Grupo de Laboratorio de SIREVA II: **[Characterization of invasive isolates of *S. pneumoniae*, *H. influenzae*, and *N. meningitidis* in Latin America and the Caribbean: SIREVA II, 2000-2005]**. *Rev Panam Salud Publica* 2008, **24**(1):1-15.
2. Oficina General de Estadística e Informática: **Información oficial de Hospitalizaciones y Mortalidad 2006. Ministerio de Salud del Perú; 2006**.
3. Ministerio de Salud del Perú: **Análisis de situación de salud del Perú - 2005. Lima: Dirección General de Epidemiología; 2006.**
4. Dirección General de Información en Salud de México: **Información dinámica: bases de datos en formato de cubo dinámico; 2008** [http://sinais.salud.gob.mx/basesdedatos/]
5. Ministério da Saúde - Sistema de Informações Hospitalares do SUS (SIH/SUS): **Morbidade Hospitalar do SUS por local de internação, Brasil, período 2007** [http://tabnet.datasus.gov.br/cgi/deftohtm.exe?sih/cnv/miuf.def]
6. Cintra O, Iwamoto M, Iwamoto M, Delcaro L, Domingos J, Paula F, Ferraz I, Matsuno A, Arruda E: **Incidence of acute otitis media (AOM) and community acquired pneumonia (CAP) in a Brazilian community level primary care services (PCS) [abstract]**. *Pediatr Infect Dis J* 2009, **28**(6):P165.
7. Gutiérrez Trujillo G, Martínez González MC, Guiscafré Gallardo H, Gómez GP, Munoz O: **Patrones de prescripción de antimicrobianos en infecciones respiratorias agudas: encuesta en la población rural / Patterns of antimicrobial-Drug prescriptions in acute respiratory infections: survey performed in the Mexican rurar area**. *Rev Fac Med UNAM* 1989, **32**(4):134-136.
8. Lopez I, Sepulveda H, Valdez I: **Frecuencia de otitis media aguda en menores de 5 anos bajo control**. *Revista Pediatria (Santiago)* 1998, **41**:21-25.
9. Organización Panamerica de la Salud: **Informe Regional de SIREVA II, 2006. Datos por país y por grupos de edad sobre las características de los aislamientos de *Streptococcus pneumoniae*, *Haemophilus influenzae* y *Neisseria meningitidis* en procesos invasores** [http://www.paho.org/Spanish/AD/THS/EV/labs_Sireva_II_2006.pdf]

10. Organización Panamerica de la Salud: **Informe Regional de SIREVA II: datos por país y por grupos de edad sobre las características de los aislamientos de *Streptococcus pneumoniae*, *Haemophilus influenzae* y *Neisseria meningitidis* en procesos invasores, 2000-2005** [http://www.paho.org/Spanish/AD/THS/EV/LABS-Sireva.pdf]
11. Instituto Nacional de Estadísticas e Informática (INEI): **Condiciones de Vida en el Perú, 2003/2004. Salud y Pobreza. Población por tenencia de seguro de salud por condición de pobreza, según tipo de seguro, área de residencia** [http://www1.inei.gob.pe/Sisd/index.asp]
12. Hospital Nacional Cayetano Heredia 2010: **Tarifario Hospitalario Basado en Costos 2007 del Hospital Cayetano Heredia** [http://www.hospitalcayetano.gob.pe/descargas/Transparencia/Planeamiento/costos/tarifarioHospitalarioHNCH.pdf]
13. Instituto Nacional de Estadísticas e Informática 2009: **Información económica, Sistema de Indices de Precios, Cuid. y Conserv. de Salud, Cuidado, Conserv. Salud y Serv. Médicos** [http://www1.inei.gob.pe/web/aplicaciones/siemweb/index.asp?id=003]
14. Berruecos P: **Cochlear implants: an international perspective - Latin American countries and Spain**. *Audiology* 2000, **39**(4):221-225.
15. Bennett JE, Sumner W, 2nd, Downs SM, Jaffe DM: **Parents' utilities for outcomes of occult bacteremia**. *Arch Pediatr Adolesc Med* 2000, **154**(1):43-48.
16. Oh PI, Maerov P, Pritchard D, Knowles SR, Einarson TR, Shear NH: **A cost-utility analysis of second-line antibiotics in the treatment of acute otitis media in children**. *Clin Ther* 1996, **18**(1):160-182.
17. Oostenbrink R, Oostenbrink JB, Moons KG, Derksen-Lubsen G, Essink-Bot ML, Grobbee DE, Redekop WK, Moll HA: **Cost-utility analysis of patient care in children with meningeal signs**. *Int J Technol Assess Health Care* 2002, **18**(3):485-496.
18. Morrow A, De Wals P, Petit G, Guay M, Erickson LJ: **The burden of pneumococcal disease in the Canadian population before routine use of the seven-valent pneumococcal conjugate vaccine**. *Can J Infect Dis Med Microbiol* 2007, **18**(2):121-127.
19. Sáez-Llorens X, Tregnaghi MW, López P, Abate H, Pósleman A, Cortes-Barbosa C, Carabajal C, Calvo A, Wong D, Falaschi A, Gómes C, Caicedo Y, Leandro A, Avakian J,

Esquivel R, Sierra A, Castrejón MM, Lepetic A, Lommel P, Hausdorff WP, Borys D, Ruiz Guiñazú J, Ortega-Barría E, Yarzábal JP, Schuerman L: **Design/setting of COMPAS: a Latin American trial evaluating the efficacy of 10-valent pneumococcal non-typable *Haemophilus influenza* protein-D conjugate vaccine (PHiD-CV)**. 29th Annual Meeting of the European Society for Pediatric Infectious Diseases (ESPID), The Hague, The Netherlands, June 7-11, 2011.

20. Tregnaghi MW, Sáez-Llorens X, López P, Abate H, Smith E, Pósleman A, Calvo A, Wong D, Cortes-Barbosa C, Ceballos A, Tregnaghi M, Sierra A, Márquez V, Rodriguez M, Troitiño M, Rüttimann R, Castrejón MM, Lepetic A, Lommel P, Hausdorff WP, Borys D, Ruiz Guiñazú J, Ortega-Barría E, Yarzábal JP, Schuerman L: **Evaluating the efficacy of 10-valent pneumococcal non-typable *Haemophilus influenzae* protein-D conjugate vaccine (PHiD-CV) against community-adquired pneumonia in Latin America**. 29th Annual Meeting of the European Society for Pediatric Infectious Diseases (ESPID), The Hague, The Netherlands, June 7-11, 2011.

21. Tregnaghi MW, Sáez-Llorens X, López P, Abate H, Smith E, Pósleman A, Calvo A, Wong D, Cortes-Barbosa C, Ceballos A, Tregnaghi M, Sierra A, Márquez V, Rodriguez M, Troitiño M, Rüttimann R, Castrejón MM, Lepetic A, Lommel P, Hausdorff WP, Borys D, Ruiz GJ, Ortega-Barría E, Yarzábal JP, Schuerman L: **Evidencia de la eficacia de la vacuna decavalente antineumococcica conjugada a la proteína D del Haemophilus influenzae no tipificable (PHiD-CV por su siglas en inglés) frente a la neumonía adquirida en la comunidad en América Latina : resultados del estudio COMPAS**. XIV Congreso Latinoamericano de Infectología Pediátrica (SLIPE), Punta Cana, Republica Dominicana, 25-28 de Mayo 2011.

22. Prymula R, Peeters P, Chrobok V, Kriz P, Novakova E, Kaliskova E, Kohl I, Lommel P, Poolman J, Prieels JP, Schuerman L: **Pneumococcal capsular polysaccharides conjugated to protein D for prevention of acute otitis media caused by both *Streptococcus pneumoniae* and non-typable *Haemophilus influenzae*: a randomised double-blind efficacy study**. *Lancet* 2006, **367**(9512):740-748.

23. Eskola J, Kilpi T, Palmu A, Jokinen J, Haapakoski J, Herva E, Takala A, Kayhty H, Karma P, Kohberger R, Siber G, Makela PH, Finnish Otitis Media Study Group: **Efficacy of a**

pneumococcal conjugate vaccine against acute otitis media. *N Engl J Med* 2001, **344**(6):403-409.
